# Deep Illumina sequencing reveals conserved and novel microRNAs in grass carp in response to grass carp reovirus infection

**DOI:** 10.1186/s12864-017-3562-4

**Published:** 2017-02-20

**Authors:** Libo He, Aidi Zhang, Pengfei Chu, Yongming Li, Rong Huang, Lanjie Liao, Zuoyan Zhu, Yaping Wang

**Affiliations:** 1 State Key Laboratory of Freshwater Ecology and Biotechnology, Institute of Hydrobiology, Chinese Academy of Sciences, Wuhan, 430072 China; 2 University of Chinese Academy of Sciences, Beijing, 100049 China

**Keywords:** Grass carp, Grass carp reovirus, microRNA, Hemorrhagic symptoms, Blood coagulation, Complement and coagulation cascades

## Abstract

**Background:**

The grass carp hemorrhagic disease caused by the grass carp reovirus (GCRV) is a major disease that hampers the development of grass carp aquaculture. The mechanism underlying GCRV pathogenesis and hemorrhagic symptoms is still unknown. MicroRNAs (miRNAs) are key regulators involved in various biological processes. The aim of this study was to identify conserved and novel miRNAs in grass carp in response to GCRV infection, as well as attempt to reveal the mechanism underlying GCRV pathogenesis and hemorrhagic symptoms.

**Results:**

Grass carp were infected with GCRV, and spleen samples were collected at 0 (control), 1, 3, 5, 7, and 9 days post-infection (dpi). These samples were used to construct and sequence small RNA libraries. A total of 1208 miRNAs were identified, of which 278 were known miRNAs and 930 were novel miRNAs. Thirty-six miRNAs were identified to exhibit differential expression when compared with the control, and 536 target genes were predicted for the 36 miRNAs. GO and KEGG enrichment analyses of these target genes showed that many of the significantly enriched terms were associated with immune response, blood coagulation, hemostasis, and complement and coagulation cascades, especially the GO term “blood coagulation” and pathway “complement and coagulation cascades.” Ten representative target genes involved in “complement and coagulation cascades” were selected for qPCR analysis, and the results showed that the expression patterns of these target genes were significantly upregulated at 7 dpi, suggesting that the pathway “complement and coagulation cascades” was strongly activated.

**Conclusion:**

Conserved and novel miRNAs in response to GCRV infection were identified in grass carp, of which 278 were known miRNAs and 930 were novel miRNAs. Many of the target genes involved in immune response, blood coagulation, hemostasis, and complement and coagulation cascades. Strong activation of the pathway “complement and coagulation cascades” may have led to endothelial-cell and blood-cell damage and hemorrhagic symptoms. The present study provides a new insight into understanding the mechanism underlying GCRV pathogenesis and hemorrhagic symptoms.

**Electronic supplementary material:**

The online version of this article (doi:10.1186/s12864-017-3562-4) contains supplementary material, which is available to authorized users.

## Background

MicroRNAs (miRNAs) are a class of small non-coding RNAs approximately 22 nucleotides in length that regulate gene expression in both animals and plants [1]. MiRNAs can interact with specific mRNA targets by binding to the 3′-untranslated region (UTR) of the mRNA targets, leading to RNA degradation or translational repression [2, 3]. Generally, miRNAs participate in a series of biological processes such as growth, development, organogenesis, tissue differentiation, regeneration, reproduction, endocrine activities, and immune responses [4, 5]. An abnormal miRNA expression pattern is often associated with a disorder in an organism or is characteristic of a disease [6–8]. Therefore, miRNAs could be used as biomarkers for some diseases.

MiRNAs are key participants involved in virus–host interactions [9]. Not only the host but also the virus could produce miRNAs that participate in virus–host interactions [10, 11]. Viral miRNAs may suppress the expression of host genes to facilitate the replication and spread of the virus; however, host miRNAs could target viral genes to limit virus replication [12, 13]. With the development of next-generation sequencing, an increasing number of miRNAs have been reported to participate in virus–host interactions, for example, miR-148a and miR-30a in cells infected by the hepatitis C virus [14], miR-34a in cells infected by the influenza A virus [15], miR-BART6-3p of the Epstein-Barr virus [16], and miR-UL112-1 of the human cytomegalovirus [17].

The grass carp (*Ctenopharyngodon idella*) has been an important aquaculture species in China for more than 60 years, accounting for more than 18% of the total freshwater aquaculture production. The production of grass carp was 5.5 million tons in 2014, making it the most highly consumed freshwater fish worldwide [18]. However, frequent outbreaks of diseases occur in the grass carp cultivation industry. Of these diseases, grass carp hemorrhage disease caused by the grass carp reovirus (GCRV) has received special attention because it causes great economic loss [19]. Although many studies on GCRV have been conducted, no effective drugs or vaccines against GCRV have been developed to date [20–26]. Moreover, the mechanism underlying GCRV infection is unknown, and the process of host–GCRV interaction is unclear.

In this study, grass carps were infected with GCRV, and spleen samples were collected before (control) and after (1, 3, 5, 7, and 9 days) infection. Deep Illumina sequencing was performed to identify the host miRNAs or possible GCRV-encoded miRNAs involved in the host–GCRV interaction. Moreover, differentially expressed miRNAs before and after GCRV infection were characterized, and the putative target genes were predicted. Our study would provide new insights into understanding the mechanism underlying GCRV infection.

## Methods

### Ethics approval and consent to participate

Animal welfare and experimental procedures were performed in accordance with the Guide for the Care and Use of Laboratory Animals (Ministry of Science and Technology of China, 2006), and the protocol was approved by the committee of the Institute of Hydrobiology, Chinese Academy of Sciences (CAS). All surgeries were performed under eugenol anesthesia, and all efforts were made to minimize suffering.

### Experimental fish

Healthy full-sib grass carps were used at 3 months of age (weight, 3–5 g; average length, 8 cm). The fish were obtained from the Guan Qiao Experimental Station, Institute of Hydrobiology, Chinese Academy of Sciences, and acclimatized in a circulating water system at 26–28 °C for 1 week before processing. The fish were fed with a commercial diet twice a day. If no abnormal symptoms were observed for 1 week, the fish were selected for further experiments.

### Virus challenge experiment and sample collection

After no abnormal symptoms were observed for 1 week, 150 grass carps were used for the virus challenge experiment. Before that, 10 fish were collected, and their spleens were sampled as an uninfected control. The remaining fish were infected with 200 μl of GCRV by intraperitoneal injection (GCRV subtype II; 2.97 × 10^3^ copies/μl). At 1, 3, 5, 7, and 9 days post-infection (dpi), 10 fish were collected, and the spleens were removed for analysis, respectively. At each time point (before and after GCRV infection), the spleen tissues of 10 fish were pooled together and used for small RNA sequencing.

### RNA isolation, library construction, and sequencing

RNA was isolated using the TRIzol reagent (Invitrogen, USA), according to the manufacturer’s protocol. RNA concentration was measured using the Qubit RNA assay kit (Life Technologies, USA), and RNA integrity was assessed with the RNA Nano 6000 assay kit (Agilent Technologies, USA). RNA of sufficiently high quality was used for library construction.

A total of 3 μg RNA per sample was used as the input material for the small RNA library. Sequencing libraries were generated using the NEBNext® Multiplex Small RNA Library Prep Set for Illumina (NEB, USA), according to the manufacturer’s recommendations, and index codes were added to attribute sequences to each sample. Briefly, the NEB 3′ SR Adaptor was directly and specifically ligated to the 3′ end of miRNA, siRNA, and piRNA. After 3′ ligation, the SR RT Primer was hybridized to the excess 3′ SR Adaptor (that remained free after the 3′ ligation reaction), which transformed the single-stranded DNA adaptor to a double-stranded DNA (dsDNA) molecule. This step is important to prevent adaptor-dimer formation; besides, dsDNAs are not substrates for ligation mediated by T4 RNA Ligase 1 and, therefore, do not ligate to the 5′ SR Adaptor in the subsequent ligation step. The 5′ end adapter was ligated to the 5′ ends of miRNA, siRNA, and piRNA. Then, first-strand cDNA was synthesized using M-MuLV Reverse Transcriptase (RNase H). PCR amplification was performed using the LongAmp Taq 2× Master Mix, SR Primer for Illumina, and index (X) primer. The PCR products were purified on an 8% polyacrylamide gel (100 V, 80 min). DNA fragments corresponding to 140–160 bp (the length of a small noncoding RNA plus the 3′ and 5′ adaptors) were obtained and dissolved in 8 μl of the elution buffer. Finally, library quality was assessed using the Agilent Bioanalyzer 2100 system and DNA High Sensitivity Chips. The libraries were sequenced on the Illumina Hiseq 2500 platform, and 125-bp single-end reads were generated.

### Data analysis

Raw data (raw reads) in the Fastq format were first processed using custom Perl and Python scripts. In this step, clean data (clean reads) were obtained by removing the reads containing poly-N, with 5′ adapter contaminants, without 3′ adapter or the insert tag, containing poly A or T or G or C, and low quality reads from raw data. Simultaneously, Q20, Q30, and GC-content of the raw data were calculated. Then, a certain range of length from the clean reads was selected to perform all the downstream analyses.

The small RNA tags were mapped to the reference sequence of grass carp [27] by Bowtie [28] with 1-nucleotide mismatch to analyze their expression and distribution. To avoid false-positive results, the small RNA reads with low expression levels (sum of reads at six time points < 10) were also discarded.

To remove tags originating from protein-coding genes, repeat sequences, rRNA, tRNA, snRNA, and snoRNA, the mapped small RNA tags were searched against the Rfam database (http://rfam.xfam.org/), and the mapped tags were ruled out. The remaining small RNA tags were used to search for known miRNAs. miRBase 21 and some known miRNAs of grass carp were used as references (mismatch ≤ 2) [29, 30], and modified software mirdeep2 [31] and srna-tools-cli were used to obtain potential miRNAs and draw the secondary structures. Custom scripts were used to obtain the miRNA counts as well as base bias on the first position of the identified miRNAs with a certain length and on each position of all the identified miRNAs.

The small RNA sequences with no homologs in miRbase but mapped to the grass carp genome and with precursors showing the RNA-loop structure were termed as novel miRNAs.

### Differential expression analysis and target gene prediction

Gene expression levels were calculated using the transcripts per million (TPM) clean tags method [32]. Calculation of expression levels and identification of miRNAs that were differentially expressed between the libraries were performed using the Edge R package based on TPM normalized counts. The settings “*P* value < 0.05” and “|log2.Fold change.normalized| > 1” were used as thresholds for judging significant differences in transcript expression.

Identification of miRNA targets was performed via computational analysis. Two miRNA target prediction algorithms, miRanda and PITA, were used to identify the target genes of the GCRV infection-related miRNAs [33, 34]. Sequences of 3′-UTRs obtained from the grass carp genome were used for the analysis. The thresholds of miRanda for candidate target sites were paring score S ≥ 200 and energy score ΔG ≤ −20 kcal/mol, where S is the sum of single-residue-pair match scores over the alignment trace and ΔG is the free energy of duplex formation from a completely dissociated state calculated using the Vienna package. The score ΔΔG ≤ −15.0 was used for PITA.

### GO and KEGG enrichment analyses of the target genes

Gene ontology (GO) enrichment analysis of the target genes was used for the target gene candidates of the differentially expressed miRNAs. All GO enrichment analyses were performed using a Cytoscape plugin, ClueGO [35]. Only categories with a low *P* value (<0.05) were considered as enriched in the network, as determined by the hypergeometric statistical test using the Benjamini and Hochberg false discovery rate correction.

The Kyoto Encyclopedia of Genes and Genomes (KEGG) database is used to provide high-level functional information on the biological systems of molecules, cells, organisms, and ecosystems, and it is particularly used for the evaluation of large-scale molecular datasets generated using genome sequencing and other high-throughput experimental approaches [36]. In this study, KOBAS software was used to test the statistical enrichment of the target genes in the KEGG pathways [37]. KEGG terms with corrected *P* < 0.05 were considered to indicate statistical significance.

### Validation of the target genes and miRNAs by using RT-qPCR

To confirm the reliability of the data obtained using Illumina sequencing, five known and five novel miRNAs were randomly selected for RT-qPCR analysis by using the oligo (dT) primer method [38]. Total RNA was isolated using the TRIzol reagent (Invitrogen, USA), according to the manufacturer’s protocol. First-strand cDNAs of miRNAs were synthesized using the miRcute Plus miRNA First-Strand cDNA Synthesis Kit (Tiangen, China). Then, the cDNAs were used as the template for qPCR with the miRcute Plus miRNA qPCR Detection Kit (Tiangen, China). RT-qPCR was performed using a fluorescence quantitative PCR instrument (Bio-Rad, USA). Each RT-qPCR mixture contained 10 μl of 2× miRcute Plus miRNA Premix, 0.4 μl of the specific forward primer, 0.4 μl of the universal reverse primer, 2 μl of the cDNA template, and 7.2 μl of ddH_2_O. Three replicates were included for each sample, and the 5S rRNA gene of grass carp was used as the internal control for normalization of gene expression. The primer sequences for the selected miRNAs are listed in Additional file 1. The program for RT-qPCR was as follows: 94 °C for 2 min, followed by 40 cycles of 94 °C for 20 s and 60 °C for 34 s. The relative expression levels were calculated using the 2^−△△Ct^ method [39]. The data were represented as the mean ± standard deviation values of three replicates.

To analyze the expression patterns of the representative target genes, 10 target genes involved in the “complement and coagulation cascades” were selected for qPCR. The primers are listed in Additional file 2. First-strand cDNAs were obtained using a random hexamer primer and the ReverTra Ace kit (Toyobo, Japan). RT-qPCR was performed using the fluorescence quantitative PCR instrument (Bio-Rad, USA). Each RT-qPCR mixture contained 0.4 μl of the forward and reverse primers (for each primer), 1 μl of the template, 10 μl of the 2× SYBR Green master mix (Toyobo, Japan), and 8.2 μl of ddH_2_O. Three replicates were included for each sample, and the β-actin gene was used as the internal control for normalization of gene expression. The program for RT-qPCR was as follows: 95 °C for 10 s, followed by 40 cycles of 95 °C for 15 s, 55 °C for 15 s, and 72 °C for 30 s. The relative expression levels were calculated using the 2^−△△Ct^ method [39]. The data were represented as mean ± standard deviation values of three replicates.

### Statistical analysis

The statistical significance between the control and treated groups was determined using one-way analysis of variance. Differences were considered significant at *P* < 0.05.

## Results

### Preliminary analysis of small RNA sequencing

At different time points before (control, 0 day) and after (1, 3, 5, 7, and 9 days) GCRV infection, the spleen tissues of 10 fish were pooled together and used for small RNA sequencing on an Illumina Hiseq 2500 platform. The six libraries showed raw read numbers of 36,743,414, 34,760,645, 31,477,182, 31,399,413, 29,264,029, and 32,534,631 (Table 1). After removing the reads containing poly-N, with 5′ adapter contaminants, without 3′ adapter or the insert tag, <18-bp reads, >30-bp reads, and low-quality reads from the raw data, the six libraries collected clean read numbers of 28,545,325, 25,745,655, 24,897,550, 25,878,410, 23,411,251, and 274,55,120. These results confirmed the adequate depth of the sequencing data and suitability for further analysis. The sequencing data of this study have been deposited in the Sequence Read Archive (SRA) at the National Center for Biotechnology Information (NCBI) (accession number: SRP093335).

**Table 1:** Preliminary analysis of the small RNA sequencing data

Samples	Total reads	Clean reads	Number of miRNAs
Control	36,743,414	28,545,325	777
T1	34,760,645	25,745,655	713
T3	31,477,182	24,897,550	651
T5	31,399,413	25,878,410	730
T7	29,264,029	23,411,251	681
T9	32,534,631	27,455,120	745

### Size distribution of small RNAs

**Fig. 1:**
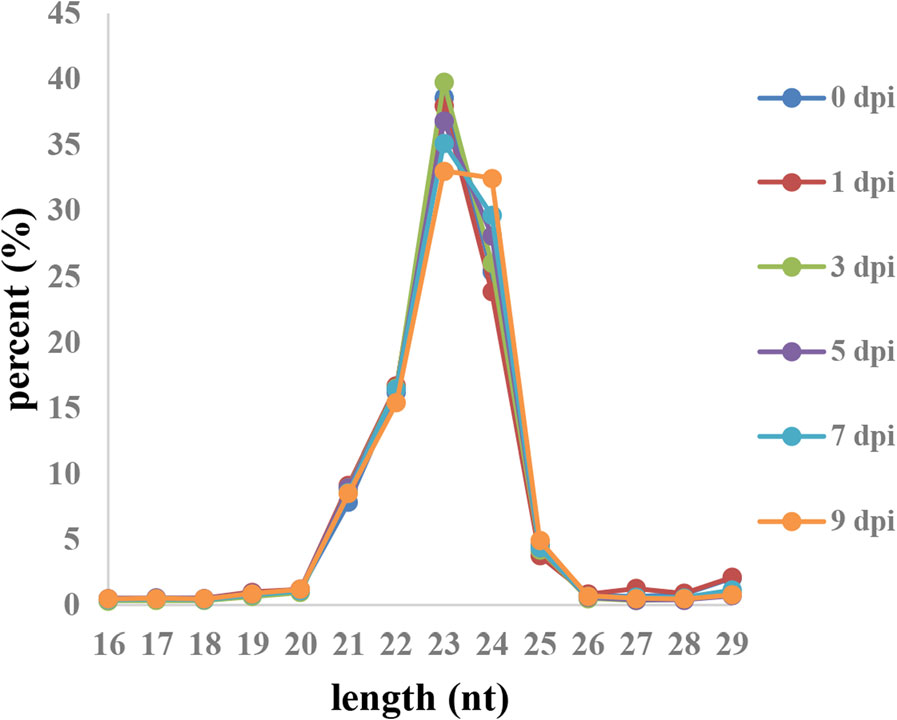
Length distributions of the sequencing reads in the six libraries. The small RNA size distribution patterns in the six libraries were examined after removing the reads containing poly-N, with 5′ adapter contaminants, without 3′ adapter or the insert tag, <18-bp reads, >30-bp reads, and low-quality reads

The small RNA size distribution patterns in the six libraries were examined. For all the six libraries, 23-length nucleotides were the most abundant, followed by nucleotides with the lengths 24 and 22 (Fig. 1). These results confirmed the homogeneity or uniformity of the sequencing data in the six libraries.

### Identification of miRNAs before and after GCRV infection

The clean reads of the six libraries were used to identify known and novel miRNAs. After a series of selections, a total of 1208 miRNAs were identified. Two hundred seventy-eight could match perfectly or find homologs in miRbase, were identified as known miRNAs, whereas the remaining 930 that found no homologs in miRbase but were mapped to the grass carp genome and had precursors with the RNA-loop structure were termed as novel miRNAs (Additional file 3). Specific, 777, 713, 651, 730, 681, and 745 miRNAs were expressed in the libraries from 0 (control), 1, 3, 5, 7, and 9 dpi, respectively (Table 1). In addition, 373 miRNAs were expressed in all the six samples (Additional file 4). Interestingly, we found that most of the known miRNAs, such as miR-143-3p, miR-21, and miR-10a-5p, showed abundant expression in all or most of the samples. However, many of the novel miRNAs showed low expression or were only expressed at some time points.

**Fig. 2:**
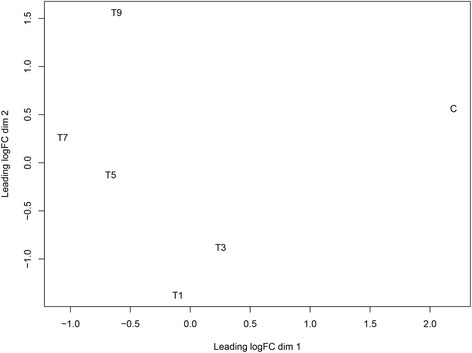
The biological coefficient of variation (BCV) of the six samples. In the plot, dim 1 showed the difference between the control and infected samples, whereas dim 2 revealed the inner difference in the infected samples

Moreover, we evaluated the correlation among the six samples. The function plotMDS affiliated with the Edge R package was used to produce a plot in which distances between the samples corresponded to leading biological coefficient of variation (BCV) between the samples [40]. The control sample separated significantly from the samples infected with GCRV (Fig. 2). Moreover, for the samples post-infection, the correlation values were proportional to the time post-infection; the sample from 9 dpi did not cluster with the samples from 1, 3, 5, and 7 dpi. These results suggested that the efficiency of the GCRV infection and difference existed in the samples post-infection.

### Differentially expressed miRNAs after GCRV infection

**Fig. 3:**
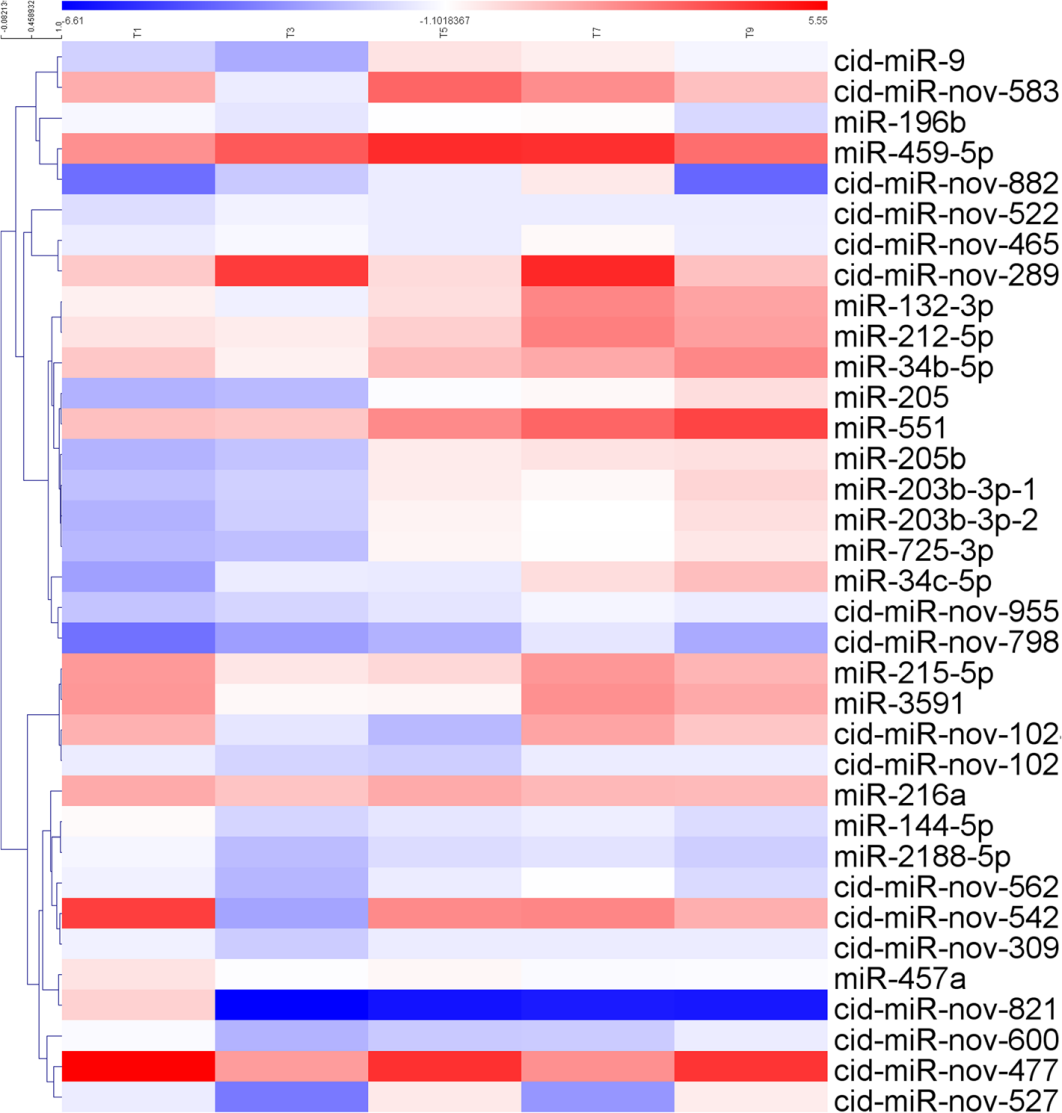
Heatmap of the differentially expressed miRNAs. Heatmap showing log_2_-fold changes in the differentially expressed miRNAs at 1, 3, 5, 7, and 9 days post-infection when compared with the control

To identify the miRNAs involved in the GCRV infection, the expression profiles of the miRNAs were examined at 0, 1, 3, 5, 7, and 9 dpi. Because some miRNAs were expressed at only one time point, to avoid false-positive results, only miRNAs that were expressed in at least three samples were selected for the differential expression analysis. Moreover, |log_2_ (fold change)| > 1 in at least three samples post-infection was set as the threshold for significant differential expression. After selection, a total of 36 miRNAs were identified as differentially expressed, of which 20 were known miRNAs and 16 were novel miRNAs. The expression pattern of the 36 miRNAs was shown in Fig. 3. Information on the differentially expressed miRNAs is listed in Additional file 5.

### Prediction of the target genes of the differentially expressed miRNAs

**Fig. 4:**
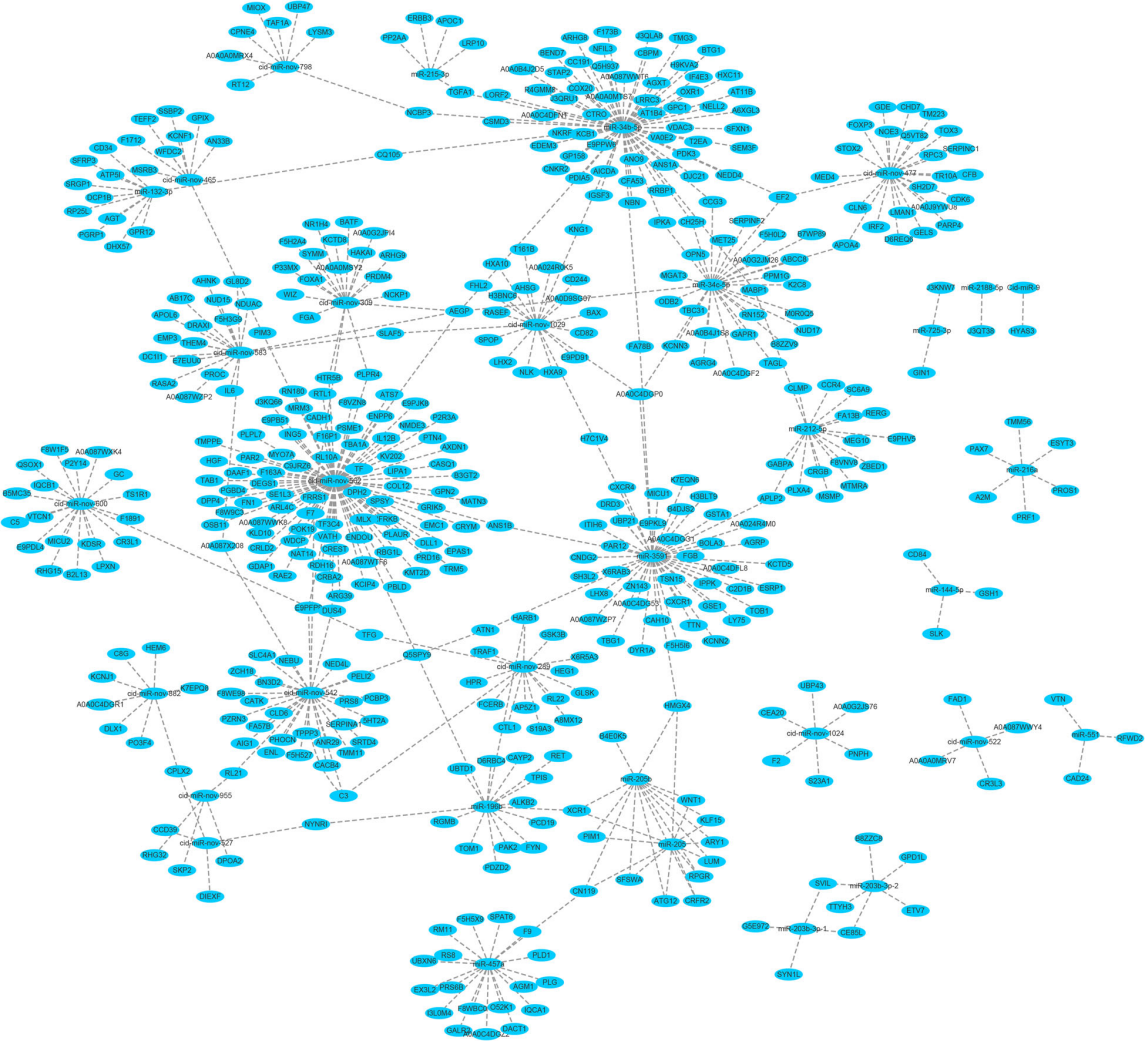
Schematic diagram of the interaction between the differentially expressed miRNAs and target genes. PITA and miRanda software were used to predict the target genes of the 36 differentially expressed miRNAs. The results show the intersection obtained by the software

PITA and miRanda software were used to predict the target genes of the 36 differentially expressed miRNAs. A total of 536 target genes were predicted for the 36 miRNAs (Additional file 6). Interestingly, we found that many genes could be targeted by one miRNA. For example, for miR-34c-5p, cid-miR-nov-562, and miR-34b-5p, 94, 86, and 65 target genes, respectively, were predicted (Fig. 4). However, for some miRNAs, such as miR-2188-5p and cid-miR-9, only one target gene was predicted. The target genes are involved in a series of biological processes. Specifically, many genes involved in the immune response, inflammatory response, and blood coagulation, such as interleukin-6 (*IL6*), interferon regulatory factor 2 (*IRF2*), complement C3 (*C3*), and grass carp reovirus induced gene 2i (*Gig2i*), were targeted by these differentially expressed miRNAs.

### GO and KEGG enrichment analyses of the target genes

GO and KEGG enrichment analyses were performed to investigate the possible roles of the target genes. For the GO enrichment analysis, most of the GO terms belonged to the biological process category, suggesting the occurrence of a series of molecular events in grass carp after GCRV infection. Many of the significantly enriched GO terms were associated with immune response and blood coagulation, such as wound healing, regulation of body fluid levels, blood coagulation, hemostasis, and coagulation. The top 10 enriched GO terms of the target genes are listed in Table 2, and details of the GO terms are shown in Additional file 7. Moreover, KEGG enrichment analysis was performed. The results showed that the enriched KEGG terms were associated with blood coagulation and response to stress, such as complement and coagulation cascades, prion diseases, African trypanosomiasis, proteoglycans in cancer, and *Staphylococcus aureus* infection. The top 10 enriched GO terms of the target genes are listed in Table 3, and details of the KEGG terms are shown in Additional file 7.

**Table 2:** Top 10 enriched GO terms for the target genes

GO ID	GO terms	Number of genes	Corrected *P* value
GO:0042060	Wound healing	31	1.02E-08
GO:0050878	Regulation of body fluid levels	30	3.24E-08
GO:0007596	Blood coagulation	29	2.77E-09
GO:0007599	Hemostasis	29	2.39E-09
GO:0050817	Coagulation	29	2.75E-09
GO:0019725	Cellular homeostasis	28	8.54E-08
GO:0032101	Regulation of response to external stimulus	28	2.22E-08
GO:0006954	Inflammatory response	27	5.36E-08
GO:0072562	Blood microparticle	24	3.67E-16
GO:0055082	Cellular chemical homeostasis	23	2.92E-06

**Table 3:** Top 10 enriched KEGG terms for the target genes

KEGG ID	KEGG terms	Number of genes	Corrected *P* value
KEGG:04610	Complement and coagulation cascades	19	1.49E-16
KEGG:05020	Prion diseases	5	0.001678
KEGG:05143	African trypanosomiasis	4	0.011383
KEGG:05205	Proteoglycans in cancer	9	0.02236
KEGG:05150	*Staphylococcus aureus* infection	4	0.039114
KEGG:04115	p53 signaling pathway	4	0.06146
KEGG:05133	Pertussis	4	0.068774
KEGG:05222	Small cell lung cancer	4	0.081635
KEGG:04672	Intestinal immune network for IgA production	3	0.084715
KEGG:05134	Legionellosis	3	0.085593

### Expression patterns of the representative target genes

**Fig. 5:**
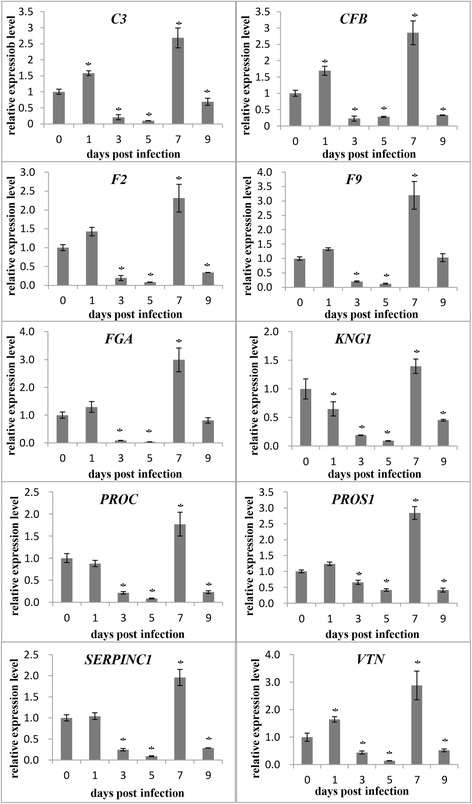
Expression patterns of 10 representative target genes. Ten representative target genes involved in “complement and coagulation cascades” were selected for qPCR to examine the expression patterns at the six time points. The relative expression levels of the target genes at different time points were calculated as the ratio of gene expression level relative to that at 0 days (control) post-infection. All data represent the mean ± standard deviation values of three replicates. Significant differences (*P* < 0.05) between the infected samples and control (0 day) are indicated with an asterisk (*)

The above-mentioned results show that many of the target genes are involved in blood coagulation or complement and coagulation cascades. Coincidently, GCRV can cause hemorrhagic symptoms in infected fish; however, the underlying mechanism is unknown. This strongly implies a correlation between these target genes and the hemorrhagic symptoms. To verify this correlation, 10 representative target genes that involved in pathway “complement and coagulation cascades” were selected for qPCR to examine the expression patterns at the six time points. These genes included *C3*, complement factor B (*CFB*), coagulation factor II (*F2*), coagulation factor IX (*F9*), fibrinogen alpha chain (*FGA*), kininogen I (*KNG1*), blood coagulation factor XIV (*PROC*), vitamin K-dependent protein S (*PROS*), antithrombin-III (*SERPINC1*), and vitronectin (VTN). Surprisingly, all the 10 selected genes shared similar expression patterns (Fig. 5). Specifically, when compared with the control (0 days), most of the genes showed slight changes at 1 dpi and were downregulated significantly at 3 and 5 dpi. However, a marked upregulation of most of these genes was observed at 7 dpi, suggesting the activation of the “complement and coagulation cascades.” This upregulation was not persistent, and it declined at 9 dpi.

### Confirmation of miRNAs by using qPCR analysis

**Fig. 6:**
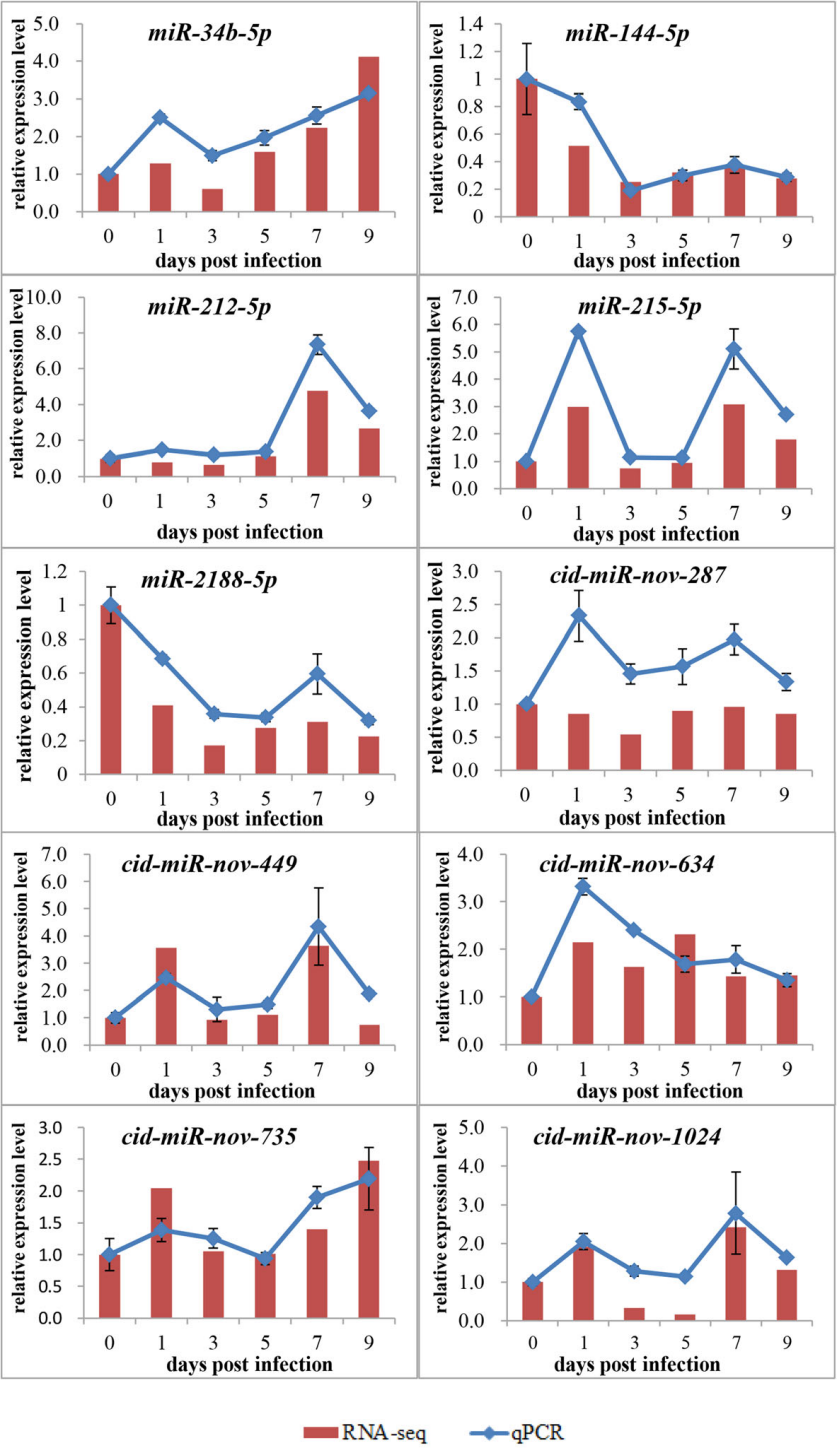
Confirmation of the RNA-seq data by using RT-qPCR. Five known and five novel miRNAs were randomly selected for RT-qPCR analysis and compared with the data obtained using RNA-seq. The relative expression levels of the miRNAs at different time points were calculated as the ratio of gene expression level (qPCR) or normalized TPM (RNA-seq) relative to that at 0 days (control) post-infection. The data are represented as mean ± standard deviation of three replicates for the qPCR analysis

Five known and five novel miRNAs were randomly selected for the RT-qPCR analysis. These miRNAs were miR-34b-5p, miR-144-5p, miR-212-5p, miR-215-5p, miR-2188-5p, cid-miR-nov-287, cid-miR-nov-449, cid-miR-nov-634, cid-miR-nov-735, and cid-miR-nov-1024. The relative expression level of the miRNAs on different dpi were calculated as the ratio of the gene expression levels relative to those at 0 dpi. For most of the examined miRNAs, the expression patterns identified using qPCR were similar to those obtained using the RNA-seq analyses, although the relative expression level were not completely consistent (Fig. 6). Therefore, the results of the qPCR analysis confirmed the reliability and accuracy of the RNA-seq data.

## Discussion

In this study, using deep Illumina sequencing, we revealed conserved and novel miRNAs in grass carp in response to GCRV infection. After a series of stringent selections, a total of 1208 miRNAs were identified at six time points, of which 278 were known miRNAs and 930 were novel miRNAs. Interestingly, the number of miRNAs found in our study was more than that in other fish, such as the Japanese flounder [12], olive flounder [41], Chinese rare minnow [42], and rainbow trout [43], but similar to that in the Japanese puffer [44]. The large number of miRNAs identified in this study may be due to the deep sequencing of the libraries. Each of the six libraries showed clean reads ≥ 20 M. Most of the known miRNAs showed abundant expression levels regardless of the time points, whereas many of the novel miRNAs showed low expression levels or were only expressed at some time points. These results suggest that deep sequencing is essential for identifying novel miRNAs with low expression level.

During virus infection, not only the host but also the virus can encode miRNAs [45]. Many studies have revealed the role of miRNAs encoded by fish viruses during pathogenesis [12, 46]. In our study, we also attempted to find the miRNAs encoded by GCRV. Some short nucleotides (20–25 nucleotides) from the clean reads were mapped perfectly to the genome of GCRV after alignment (data not shown). However, no precursor sequences with the RNA-loop structure were found for these short nucleotides. Therefore, these short nucleotides were not miRNAs encoded by GCRV. Previous studies have suggested that only viruses from the families *Alpha-herpesvirinae, Beta-herpesvirinae, Gamma-herpesvirinae, Polyomaviridae, Ascoviridae, Baculoviridae, Retroviridae,* and *Adenoviridae* can encode miRNAs or miRNA-like molecules [45, 47]. These viruses are DNA viruses or have a period with a DNA genome. For RNA viruses, no viruses that encode miRNAs have been reported; only engineered RNA viruses can express biologically active miRNAs or miRNA-like molecules [48, 49]. Thus, GCRV and other reoviruses may not have the ability to encode miRNAs.

Using a rigorous standard for the selection of differential expression, only 36 miRNAs were identified to exhibit differential expression. However, a total of 536 target genes were predicted for the 36 miRNAs. Interestingly, many of the genes could be targeted by one miRNA, such as miR-34c-5p, cid-miR-nov-562, and miR-34b-5p. One miRNA targeting many genes may suggest that the miRNA may participate in a series of biological processes. Previous studies have also shown that miR-34b-5p and miR-34c-5p are involved in many biological events [50–53]. GO and KEGG enrichment analyses were performed for the 536 target genes. The results showed that a lot of GO terms or KEGG pathways were enriched. Specifically, the GO term “blood coagulation” and pathway “complement and coagulation cascades” were the most significantly enriched. Coincidently, GCRV can cause hemorrhagic symptoms in infected fish. Therefore, we hypothesized a correlation between the hemorrhagic symptoms and these GO terms or pathways. qPCR analysis of the representative genes revealed that most of the genes were upregulated markedly at 7 dpi, suggesting the activation of “complement and coagulation cascades.” This result was strongly consistent with that of our previous study ([54], He et al., unpublished data), implying the facticity and reliability of the experiment. Activation of the complement cascade can lead to endothelial and blood-cell damage, resulting in platelet activation and aggregation, hemolysis, and prothrombotic and inflammatory changes [55, 56]. Finally, the hemorrhagic symptoms could due to the overactivity of the complement cascade. However, the upregulation was not persistent, and it declined at 9 dpi. This may be because death was observed at 9 dpi; the complement system was deactivated at this time point.

## Conclusions

In conclusion, conserved and novel miRNAs in grass carp in response to GCRV infection were identified. Thirty-six miRNAs were identified to exhibit differential expression, and they targeted 536 genes. Many of the target genes were involved in immune response, coagulation, hemostasis, and complement and coagulation cascades. qPCR analysis of the representative genes suggested that the pathway “complement and coagulation cascades” was activated strongly, leading to endothelial-cell and blood-cell damage and hemorrhagic symptoms. The present study provides a new insight into understanding the mechanism underlying GCRV pathogenesis and hemorrhagic symptoms caused by GCRV.

## Additional files

Additional file 1: Primer sequences for qPCR analysis of 10 miRNAs. (DOCX 16 kb)
Additional file 2: Primer sequences for qPCR analysis of 10 target genes. (DOCX 16 kb)
Additional file 3: Detailed information on 1208 miRNAs. (XLSX 278 kb)
Additional file 4: Detailed information on the 373 miRNAs expressed in all the six samples. (XLSX 155 kb)
Additional file 5: Detailed information on the 36 differentially expressed miRNAs. (XLSX 23 kb)
Additional file 6: Detailed information on the 536 target genes. (XLSX 36 kb)
Additional file 7: Detailed information on the GO terms and KEGG pathways of the 536 target genes. (XLSX 265 kb)

## Abbreviations

BCV: Biological coefficient of variation
C3: Complement C3
CFB: Complement factor B
F2: Coagulation factor II
F9: Coagulation factor IX
FGA: Fibrinogen alpha chain
GCRV: Grass carp reovirus
Gig2i: Grass carp reovirus induced gene 2i
GO: Gene ontology
IL6: Interleukin-6
IRF2: Interferon regulatory factor 2
KEGG: Kyoto Encyclopedia of Genes and Genomes
KNG1: Kininogen I
NCBI: National Center for Biotechnology Information
PROC: Blood coagulation factor XIV
PROS: Vitamin K-dependent protein S
RT-qPCR: Real-time quantitative PCR
SERPINC1: Antithrombin-III
SRA: Sequence Read Archive
TPM: Transcripts per million
UTR: Untranslated region
VTN: Vitronectin
