# Supplementary material for: Deep Illumina sequencing reveals conserved and novel microRNAs in grass carp in response to grass carp reovirus infection
Source: BMC Genomics. 2017 Feb 20;18:195. doi: 10.1186/s12864-017-3562-4 (PMC5319172; doi:10.1186/s12864-017-3562-4)
Supplement: Additional file 1: — Primer sequences for qPCR analysis of 10 miRNAs. (DOCX 16 kb) [file 12864_2017_3562_MOESM1_ESM.docx]

**Additional file 1**

**Primer sequences for qPCR analysis of 10 miRNAs**

| **miRNAs** | **Forward primer sequences (5’-3’)** |
| --- | --- |
| miR-34b-5p | GCTGGCAGTGTCTTAGCTGGTTGT |
| miR-144-5p | GCGCGGATATCATCGTATACTGTAAGT |
| miR-212-5p | GCGACCTTGGCTCTAGACTGCTTACT |
| miR-215-5p | GCGATGACCTATGAATTGACAGCC |
| miR-2188-5p | GCAAGGTCCAACCTCACATGTCCT |
| cid-miR-nov-287 | CTCGGACCAGCATAAACCAGCTC |
| cid-miR-nov-449 | GCACAGGAAATGTTCTGAAGAAGCT |
| cid-miR-nov-634 | CGCTCAGTAACTGGTATCTCTCCCTGT |
| cid-miR-nov-735 | GCCGTCTGTAATGAATGTCAAGACCT |
| cid-miR-nov-1024 | GCGATCCGTCACGTGTGACCT |
| 5S rRNA | TCTCGGAAGCTAAGCAGGGTCG |
