# Supplementary material for: Deep Illumina sequencing reveals conserved and novel microRNAs in grass carp in response to grass carp reovirus infection
Source: BMC Genomics. 2017 Feb 20;18:195. doi: 10.1186/s12864-017-3562-4 (PMC5319172; doi:10.1186/s12864-017-3562-4)
Supplement: Additional file 2: — Primer sequences for qPCR analysis of 10 target genes. (DOCX 16 kb) [file 12864_2017_3562_MOESM2_ESM.docx]

**Additional file 2**

**Primer sequences for qPCR analysis of 10 target genes**

| **Target genes** | **Primer sequences (5’-3’)** | **efficiency** |
| --- | --- | --- |
| *C3* | ATGGTTCGCAAACACTCCTCAG  ACAGGTACTTGGCTTCTATGTCAACT | 94.7% |
| *CFB* | ATGACTGGAGGGAGCTTTGTTC  GTTTTTCGGGTGCTGGTCTTT | 101.0% |
| *F2* | ACCCTCCGCCACCTCCTAT  GGGACCCTCCATATCTCCATCT | 104.0% |
| *F9* | CACACTGTAGCGGAGTGGCATAT  CGCGTTCTTGAGCAGGTTCT | 103.1% |
| *FGA* | GAAGGGTCCACTGCCGAATC  TGGGTATCAGAGCCAAGCCT | 92.9% |
| *KNG1* | TGAAGGTGCTCAACTGGCTCT  AGTAACTCTTGACCTGCTTCTGTAAAC | 101.3% |
| *PROC* | ATTGGAGCGGGAGTGTAAGGA  CAGAGTTGCAGGTGCAGGAGTA | 91.0% |
| *PROS1* | CTCGTGGTTCATGCTGGGTT  CAGTTTGTCGTTGACGGGTG | 96.8% |
| *SERPINC1* | CAACAGCCACTTCGCCCTC  GGCGAAGAAGTAGTGAACCTGG | 97.1% |
| *VTN* | AAAACATTCAAGGGCATCACAA  CCAATCCAAAGTCTTCCCAAAA | 91.9% |
| *β-actin* | AGCCATCCTTCTTGGGTATG  GGTGGGGCGATGATCTTGAT | 99.2% |
